# Financial distress among women with advanced or metastatic breast cancer in Germany: secondary analysis of prospective ePROCOM data of a two-center cohort

**DOI:** 10.1007/s00404-026-08523-y

**Published:** 2026-07-15

**Authors:** Joachim Graf, Harald Abele, Markus Wallwiener, Eva J. Kantelhardt, Sara Y. Brucker, Elisabeth Simoes

**Affiliations:** 1 Section of Midwifery Science, Institute of Health Sciences, University Hospital Tübingen, Calwer Strasse 7, 72076 Tübingen, Germany; 2 Department for Women’s Health, University Hospital Tübingen, Tübingen, Germany; 3 Department of Gynecology, Martin Luther University Halle-Wittenberg, Halle (Saale), Germany; 4 Global & Planetary Health Working Group, Center of Health Sciences, Medical Faculty of the Martin Luther University Halle-Wittenberg, Halle (Saale), Germany

**Keywords:** Breast cancer, Metastatic, Financial burden, Poverty, ePROCOM, Secondary analysis

## Abstract

**Key message:**

In this study, half of all female patients with advanced and metastatic breast cancer reported experiencing financial difficulties. This highlights the need for gynecology and oncology specialists to also address patients’ socio-medical needs. Electronic patient-reported outcomes (ePRO) must be used to identify such areas of concern.

**Abstract:**

**Purpose:**

The aim was to investigate whether and to what extent female patients with advanced or metastatic breast cancer who are undergoing chemotherapy report financial difficulties due to their physical condition and/or the effects of medical treatment in relation to the German reference cohort.

**Methods:**

A secondary data analysis was performed using data collected prospectively as part of the ePROCOM study. A total of *n* = 168 female patients who had answered questions regarding financial implications of the EORTC QLQ-C30 questionnaire were included in the analysis. Unpaired t tests (Welch) and Chi-square tests as well as multiple regressions were conducted. A bilateral *p* value of < 0.05 was considered statistically significant in all analyses (alpha = 0.05).

**Results:**

The prevalence of financial distress was 50.0% (95%-CI 42.4%; 57.6%), defined as a response of at least 2 (“a little”) on a 4-point Likert scale. Between the reference cohort scored 4.8 and the breast cancer cohort scored 28.8, there was a significant difference of 24 ± 5.19 score points (*p* < 0.001; 95%-CI 18.81, 29.19) in financial burden. Overall, the regression model explained 27.7% of the total variance in financial burden through HRQoL, age, and civil status (*R*^2^ = 0.277).

**Conclusion:**

Women with advanced or metastatic breast cancer reported substantially higher financial burden than the female German reference population. Lower HRQoL, younger age, and living alone were associated with greater financial impact. These findings support routine assessment of financial distress in those patients and suggest that ePRO-based screening may help identify patients in need of early social-medical support.

## What does this study adds to the clinical work

**Table:** 

The results of this study show that female patients with advanced and metastatic breast cancer are frequently affected by financial consequences. In clinical practice, this highlights the need for the routine social histroy that should also include economic aspects.	

## Background

### Breast cancer: burden of disease

It is estimated that by 2030, the global incidence of female breast cancer will reach approximately 2.64 million cases, with an annual mortality rate of 1.7 million women [1]. In Germany, approximately 75,000 women are newly diagnosed with breast cancer each year. Statistically, one in eight women in this country will receive a breast cancer diagnosis during her lifetime [2–5]. The main risk factors for the disease are age, hormonal factors (early menarche, late menopause), genetic predisposition (BRCA1/2 mutations), and lifestyle factors, such as being overweight after menopause, lack of physical activity, and alcohol consumption [6, 7]. Epidemiologically, breast cancer is primarily a disease of middle and older age: 15% of women are diagnosed before the age of 50 only, while 29% are diagnosed after the age of 75; the average age at diagnosis for breast cancer in Germany is approximately 65 years [2–5]. However, cases of breast cancer in young women are also on the rise, such as pregnancy-associated breast cancer (PABC), which statistically occurs in 1 in 3,000 women in the context of pregnancy, childbirth, and the postpartum period [8]. PABC poses a challenge for women’s health, obstetrics, and oncology due to the poorly understood tumor biology and the poor prognosis [9]. As a result of breast cancer screening, approximately 80% of tumors are diagnosed in early stages (I or II) [4, 5], offering good treatment options and a high probability of survival [10]. While the 5-year survival rate in Stage I is nearly 100%, overall survival is significantly poorer for patients with metastatic disease: here, the 5-year survival rate is only around 30% [11, 12]. In a recent study from Germany involving 1000 patients with metastatic disease, the 5-year survival rate was as low as 17% [13]. Due to disease- and treatment-related burdens, significant impairments in health-related quality of life (HRQoL) are also to be expected [14]. In Germany, the proportion of metastatic breast cancer accounts for approximately 10% of the total disease burden; accordingly, approximately 7500 new cases can be expected annually [2–5].

### ***Social welfare implications of breast cancer***

Metastatic breast cancer is also of relevance from a social welfare perspective, since a wide range of care and support services must be coordinated alongside inpatient and outpatient treatment options. Clinical social medicine plays a particularly important role in this context in promoting patient participation: This also refers to support for participation, given that the likelihood of navigating the healthcare system successfully—which influences the prognosis—depends on many social and sociodemographic factors, which thus become relevant to the prognosis themselves [15]. Due to its importance, breast cancer has already been included in the German list of national health goals, which aims to “reduce mortality and improve quality of life.” In July 2015, these health goals were enshrined in law by the Prevention Act in Sec. 20 para. 3 No. 2, Book V of the German Code of Social Law (Sozialgesetzbuch [SGB]) [4]. To live with dignity, breast cancer patients and long-term survivors are sometimes dependent on social system support services for a lifetime, particularly in cases of metastasis [16]. The aim of the social welfare system here should be to mitigate social inequality resulting from illness. It should be noted that determinants of health have a multifaceted influence on the course of the disease—a lower socioeconomic status is associated with a higher risk of developing breast cancer and a poorer prognosis [17–19]. Jansen et al. [20] were also able to demonstrate this explicitly for Germany: Patients were assigned a socioeconomic status according to the district of residence at diagnosis. The study shows that women with breast cancer from lower socioeconomic backgrounds have a reduced survival time.

Public health studies indicate that diseases increase social inequality and pose a risk of poverty [21–23]. This also applies to breast cancer: there is substantial evidence regarding the economic challenges faced by survivors, according to the findings of a review [24]. This is especially true for survivors of metastatic breast cancer [16]. In Germany, it has been demonstrated that breast cancer significantly increases the risk of poverty: In a longitudinal population-based study, significantly more long-term breast cancer survivors reported financial difficulties both 5 years (19.2% vs. 7.9%, *p* < 0.0001) and 10 years after the end of therapy (22.0% vs. 9.1%, *p* < 0.0001), compared to the age-matched control group without disease burden [25]. The long-term effects of the disease and treatment, which also influence the possibility of returning to work (the planning of which is one of the core socio-medical roles of doctors in Germany [26]), are not adequately taken into account in the health and social services system [15].

### Aims

To what extent financial constraints in Germany exist not only for long-term survivors but also for patients currently undergoing treatment was analyzed in this study using a cohort of female patients with advanced or metastatic breast cancer. The aim of this study was to investigate whether and to what extent female patients with advanced or metastatic breast cancer who are currently undergoing chemotherapy report self-perceived financial difficulties associated with their physical condition and/or the effects of medical treatment.

## Methods

### Study design

A secondary data analysis was performed using data collected prospectively as part of the ePROCOM study conducted in Tübingen and Heidelberg from *n* = 202 patients with advanced/metastatic breast cancer (stages III and IV) who were currently undergoing chemotherapy. This study focused on willingness to use electronic tools for measuring patient-reported outcomes (PROs). Recruitment and study methodology are described in the original papers [27–29]. In the present work, previously unpublished data were analyzed. All patients who had answered the question regarding financial implications in the original survey were included. The original study had received approval from the relevant ethics committee (Project Nos. 196/2015B02 and 089/2015B02).

### Patients and data collection

A total of n = 168 female patients were included in the analysis. In the original study, these patients had also completed the EORTC QLQ-C30 questionnaire, which is used as a PRO to measure HRQoL in cancer patients [30]. This 30-item questionnaire includes a question asking whether their physical condition or medical treatment has caused financial difficulties. Patients rate this question on a four-point scale (1 = not at all; 2 = a little; 3 = quite a bit; 4 = very much) [31]. The question was evaluated in accordance with the questionnaire’s guidelines, in which a score was calculated: *Score* = *{(RawScore − 1)/range}* × *100* [32]. A score of 0 represents no financial impact at all, while a score of 100 indicates the greatest possible financial impact [32]. Furthermore, data were available to conduct the regression analysis [in which the EORTC QLQ C-30 dimension on financial impact served as the dependent variable (DV)], including the HRQoL score from the same questionnaire as well as the social parameters of age, educational level, and civil status [27–29]. To assess the degree of disease- and treatment-related financial burden, the score was compared with data from the healthy reference population in Germany without breast cancer [33]. As part of the regression analysis, *n* = 24 patients were excluded due to missing data on the socioeconomic parameters of age, educational level, or civil status. These were Missing Completely at Random (MCAR) data, which is why listwise deletion was applied.

### Data analysis

First, the data were analyzed descriptively. Unpaired t tests (Welch) and Chi-square tests were performed to compare the cohort analyzed here with the reference population. Multiple regressions were conducted to examine whether the degree of financial burden depends on HRQoL, age, educational level, and civil status. Here, inclusion (ENTER) was selected as the method for variable inclusion. The prerequisites (including linearity of the relationship, linearity of the coefficients, homoscedasticity, and others) were checked in advance. The effect size of R^2^ was also calculated in this process as follows: ${f}^{2}= \frac{{R}^{2}}{1-{R}^{2}}$. According to Cohen (1992), a small effect was assumed at *f*^2^ = 0.02, a moderate effect at *f*^2^ = 0.15, and a strong effect at *f*^2^ = 0.35 [34]. The Cook's distance was also calculated for the individual items to identify any outliers as part of a sensitivity analysis. In line with the literature, Cook's distances > 1 were defined as influential cases, the exclusion of which would affect the variance of the regression model [35]. A bilateral *p* value of < 0.05 was considered statistically significant in all analyses (alpha = 0.05). The statistical analyses were performed using IBM SPSS 31.0.

## Results

### General characteristics and HRQoL

Table 1 compares the general characteristics of the participants included in the study with those of the female reference cohort in Germany [33]. The mean age of the patients was 52.9 ± 11.5 years. Compared to the reference group, the patient group were significant older, and had a significantly higher proportion of individuals with at least 12 years of schooling and a significantly lower proportion of individuals who reported living alone. HRQoL was 14.8 score points lower in the patient group than in the reference cohort (*p* < 0.001).

**Table 1:** General characteristics and HRQoL

Variables	Breast cancer patients (*n* = 168)^1^	Female reference population in Germany (*n* = 1,309) [33]	t	χ^2^	*p *value
Age: mean (SD)	52.9 (11.5)	50.2 (17.4)	2.68		0.008
*Civil status: n (%)*				29.0	< 0.001*
Living alone	35 (22.3)	586 (44.8)			
Married/co-habiting	122 (77.7)	723 (55.2)			
*Education (years): n (%)*				52.2	< 0.001*
≤ 9 years	42 (28.4)	525 (40.1)			
10–11 years	44 (29.7)	561 (42.9)			
≥ 12 years	62 (41.9)	223 (17.0)			
HRQoL-Score: Mean (SD)	59.7 (22.8)	74.5 (19.4)	8.05		< 0.001*

*HRQoL* health-related Quality of Life; SD = standard deviation; statistically significant at α = 0.001 (Bonferroni correction of α = 0.05 for multiple testing based on a total of 5 tests conducted in Tables 1 and 2)

^1^N = 168 refers to the number of patients who provided information regarding financial burdens in the EORTC QLQ C-30. For education, information was available for *n* = 148 patients, and for the civil status subcategory, for n = 157 patients

### Financial impact

The two cohorts showed a significant difference of 24 ± 5.19 score points (*p* < 0.001, 95%-CI 18.81, 29.19) in financial burden: On a scale ranging from 0 (no financial burden) to 100 (highest financial burden), patients had an average score of 28.8, while the reference cohort scored 4.8 (Table 2).

**Table 2:** Financial burden

Variables	Breast cancer patients (*n* = 168)	Female reference population in Germany (*n* = 1,309) [33]	t	*p *value
EORTC QLQ-C30—Financial impact score: Mean (SD)	28.8 (33.6)	4.8 (16.3)	9.1	< 0.001*

*SD* standard deviation

^*^statistically significant at α = 0.001 (Bonferroni correction of α = 0.05 for multiple testing based on a total of 5 tests conducted in Tables 1 and 2)

**Fig. 1:**
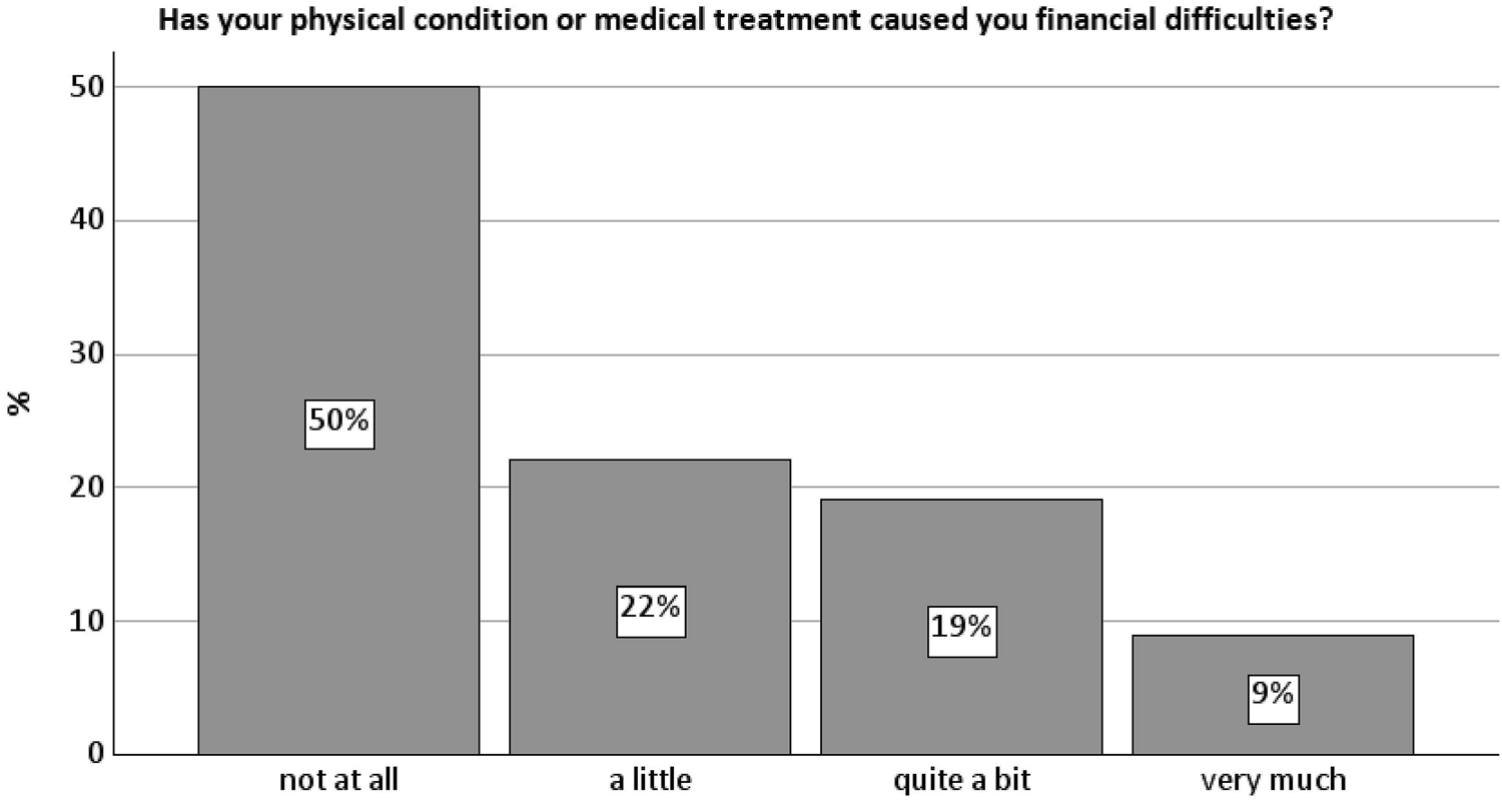
Reported financial burden (item 28 of the EORTC QLQ-C30 questionnaire), n = 168

The prevalence of financial distress was 50.0% (95%-CI 42.4%; 57.6%) in the cohort of breast cancer patients analyzed here, defined as a response of at least 2 ("a little") on a 4-point Likert scale. Overall, 22% reported a little, 19% quite a bit, and 9% very much financial distress (see Fig. 1).

### Regression analysis

The multiple regression model was significant as a whole (F(5138) = 10.56, *p* < 0.001). Table 3 examines the significance of the regression coefficients. A significant effect was found for the variable HRQoL-Score (*t* = −5.41, *p* < 0.001), Age (*t* = −2.03, *p* = 0.044), and civil status (*t* = −2.46, *p* = 0.015). A one-point increase in the HRQoL score was associated with a reduction in financial burden of 0.64 score points. With regard to age, each additional year of age was associated with a reduction in financial burden of 0.46 score points. Individuals living alone had a significantly higher score. Educational level did not influence the extent of financial burden.

**Table 3:** Significance of the regression coefficients

	Non-standardized coefficients		Standardized coefficients				Collinearity statistics		Cook distance
Model (DV = Financial Impact Score)	Regression coefficient B	SE	Beta	t	p value	95%-CI	tol	VIF	mean (min; max)
(Constant)	102.28	14.84		6.89	< 0.001*	72.94; 131.63			0.008 (0.0001;0.103)
EORTC QLQ-C30: HRQoL-Score	−0.66	0.12	-0.42	−5.41	< 0.001*	−0.90; −0.42	0.89	1.13	
Age	−0.45	0.22	-0.15	−2.03	0.044*	−0.90; −0.12	0.94	1.06	
Civil status: married/co-habiting^1^	−14.94	6.29	-0.18	−2.38	0.019*	−27.37; −2.51	0.90	1.12	
Education: ≤ 9 years^2^	11.17	6.70	0.15	1.69	0.092	−1.87; 24.21	0.70	1.43	
Education: ≥ 12 years^2^	−0.37	6.15	-0.005	−0.06	0.952	−12.53; 11.80	0.66	1.52	

*CI* confidence interval, *DV* dependent variable, *SE* standard error, *tol* tolerance, *VIF* variance influence factor

^*^statistically significant at α = 0.05

^1^Civil status: In the regression model, “living alone” was coded as “0” (= reference category), and “married/co-habiting” was coded as “1”. Variable categories were coded as dummy variables, “living alone” was excluded from the regression model

^2^Education: In the regression model, “ ≤ 9 years” was coded as “0” (= reference category), “10–11 years” as “1,” and “ ≥ 12 years” as “2”, encoded as dummy variables “, 10–11 years” was excluded from the regression model

Table 4 shows the model summary. Overall, this model explained 27.7% of the total variance in financial burden through HRQoL, age, and civil status (*R*^2^ = 0.277) with a large effect size (*f*^2^ = 0.383). No outliers were found for the Cook distance in the sensitivity analysis.

**Table 4:** Model summary

Model	R	R^2^	Adjusted R^2^	SE	Durbin–Watson statistics	f^2^
1	0.526	0.277	0.251	29.647	1.658	0.383

*SE* standard error

## Discussion

The aim of this study was to investigate whether breast cancer patients with advanced or metastatic disease experience disease- and treatment-related financial burdens. In a cohort consisting of a total of *n* = 168 patients, the prevalence of financial problems was 50%. A significantly higher burden was observed compared to the reference population without the disease; the regression model revealed associations with HRQoL, age, and civil status.

Other studies have also demonstrated that financial burden plays a role among breast cancer patients and survivors, and that there is a correspondingly increased risk of poverty [16, 24]. In a systematic review of high-income countries, Yin et al. [36] found a prevalence of 39.3% for financial toxicity in breast cancer. Ehsan et al. [37] determined a pooled rate of 35.3% in a review of high-income countries. Vancoppenolle et al. [38] examined the financial toxicity and socioeconomic impact of cancer in Europe without limiting their analysis to breast cancer and found that 56% of patients reported income loss and 86% additional treatment-related expenses. For Germany, Fabian et al. (2022) found a prevalence of financial burden among cancer patients (not limited to breast cancer) of 33% based on question 28 of the EORTC QLQ C-30 questionnaire (i.e., analogous to the present study) [39]. For breast cancer patients in Germany currently undergoing radiotherapy, Tetzlaff et al. (2026) determined a prevalence of financial difficulties of 39.1% using the EORTC QLQ C-30 questionnaire [40]. However, the studies mentioned did not stratify by tumor stage. Our study is the first to explicitly quantify the financial burden for stage III and IV female breast cancer patients in Germany and to determine a corresponding burden of 50% for this patient group. This means that every second patient with a severe course of the disease, sometimes requiring palliative care and with a correspondingly limited life expectancy, is additionally confronted with questions regarding the financing of their livelihood. We were able to show that this applies in particular to younger patients and to those living alone. As outlined in the introduction to this study, breast cancer cases are increasing among younger patients [8, 9]. Vancoppenolle et al. [38] also identified young age and living alone as key predictors in their regression analysis. Among breast cancer survivors in Germany, having an immigrant background was also a predictor of a higher financial burden [41].

This study also demonstrated that patients with lower HRQoL are more likely to report a higher financial burden; however, other studies should investigate whether the financial burden further worsens HRQoL or whether low HRQoL is associated with reduced ability to participate, which in turn leads to an increase in the financial burden, because this causal relationship could not be tested in this study due to its cross-sectional design. As part of a secondary data analysis in Italy, Perrone et al. (2016) found that financial burden was a predictor of a higher probability of a poorer overall quality-of-life response in study data on lung, breast, or ovarian cancer [42].

Another unique feature of this study is that the financial burden on female cancer patients was explicitly compared with that of a healthy reference cohort of the same age and gender, in order to highlight the impact of the disease in a public health context. This distinguishes the present study from the other studies cited here, which assessed the financial burden solely within the cancer cohort [36–42].

This study had several limitations that restrict its generalizability. The study was conducted as a secondary data analysis in which existing study data were evaluated [27–29]. Consequently, neither recruitment, inclusion, nor the study's progress could be controlled; accordingly, neither selection bias nor performance bias can be ruled out. Because the original study included patients with stage III and stage IV disease, but the raw data did not specify which patients belonged to which stage, it was not possible to perform stage-specific stratification here, although it must be assumed that patients with stage III and stage IV disease also differ significantly from one another in terms of the extent of their financial burden. Similarly, biases may have arisen because the quality of documentation could not be verified. Another limitation is that the sample may not be representative of the population. The comparison of the general data with those of the reference population [33] revealed significant differences in educational level and civil status. In the present study, there were significantly more patients with a higher educational level and in a stable relationship in relation to the reference cohort, which means that they had more favorable sociodemographic conditions. It is therefore conceivable that patients with stages III and IV breast cancer in the general population exhibit an even higher prevalence of financial burden. Another limiting factor was that the data on the reference cohort were older (published in 2014) than the data on financial burdens published as part of the original ePROCOM study (collected in 2017). Unfortunately, there are no more recent publications on the reference population. It can be assumed that surveys from 2026 could accordingly identify an even higher financial burden (among other reasons, due to the financial impact on many households from current crises—such as the COVID-19 pandemic and wars in Ukraine and the Middle East) [43]. In the present study, only the financial burden reported in the EORTC QLQ-C30 was taken into account, but not direct determinants of financial distress, such as household income, employment status at the time of diagnosis, and insurance status (public or private health insurance). A higher R^2^ is to be expected if these variables are taken into account in future studies. Furthermore, this study included only individuals who were treated exclusively at university medical centers, which tend to be utilized by patients from higher socioeconomic backgrounds [15]. This also suggests that the effects identified here could be even greater in the general population. Finally, a limiting factor was that when comparing individual parameters with the reference cohort [33], independent t tests had to be used without verifying the assumptions in the reference cohort, because only means, standard deviations, and sample sizes were available as usable values, rather than raw data. In the study assessing HRQoL in the healthy reference population, there was also a relatively low response rate, which is why measured effects may have been over- or underestimated [33]. Another limitation arose from the study design: The secondary data analysis conducted followed a cross-sectional design, meaning that no causal conclusions could be drawn. Although the regression model used HRQoL as a predictor of financial burden, this was a methodological choice rather than a causal claim.

Future research should, therefore, focus on determining the financial burden—differentiated by cancer stage—in large cohorts of both cancer survivors [25] and cancer patients currently undergoing chemotherapy or radiotherapy, ideally using longitudinal data, in order to derive recommendations for health policy. Another important consideration in this regard is when, how, or whether changes occur in how individuals cope with financial burdens as the disease progresses. These would serve as starting points for targeted reforms and individualized support programs. The current reform aimed at containing costs in Germany’s statutory health insurance system calls for reducing both the duration and the amount of sickness benefits, as well as increasing copayments for medications [44]. This will particularly affect cancer patients who, as long-term survivors and chronically ill individuals, must cope with limitations and side effects for the rest of their lives and, as patients with metastatic disease, require support from the social welfare system for the entirety of their remaining lifespan. Already today, cancer in Germany accounts for the second-longest period of work disability per case according to ICD-10 diagnostic chapters [45]. As outlined in the introduction, breast cancer will continue to be of high epidemiological significance in the future. Nearly half of all patients are younger than 65 at diagnosis [2–8] and are thus of working age; consequently, when they fall ill, they are dependent on healthcare benefits (including sick pay)—benefits that are set to be cut as part of current reform efforts [44].

Our findings are consistent with the notion that illness can contribute to financial strain and may increase poverty risk in Germany for female breast cancer patients with advanced or metastatic disease—not only for long-term survivors [25, 41], but also for those currently undergoing treatment, whose focus should be on recovery. Paragraph 2a, Book V of the German Social Code (Sozialgesetzbuch = SGB) states that the health and social care system must “take into account the special needs of people with disabilities and chronic illnesses” [46]. Social medicine's core principles include “poverty makes people sick,” meaning “socioeconomic status influences health,” and “illness makes people poor,” meaning “health influences socioeconomic status.” The second of these has long been given less attention in Germany, likely because the establishment of statutory health insurance more than 120 years ago seemed to have largely resolved the problem of “illness leads to poverty.” Does a shift away from this objective of statutory health insurance appear to be emerging as reform efforts intensify? It falls as well to the design of the health care system as to the medical disciplines responsible for cancer treatment to strengthen patients’ opportunities for participation and improve the chances of navigating the healthcare system successfully [15].

Our findings also point toward a broader role of ePRO-based monitoring in advanced breast cancer care. Financial distress could be assessed together with HRQoL, symptom burden, and, in future studies, time toxicity. This would allow clinicians to identify not only physical deterioration but also social and practical strains that may affect daily life and treatment participation. Digital screening tools could use predefined thresholds or longitudinal changes to prompt referral to social services, psycho-oncology, rehabilitation counseling, occupational support, or financial navigation. In the longer term, transparent AI-supported risk models may help identify patients at increased risk of financial toxicity by combining PRO data with sociodemographic and clinical information. Such models should be designed as decision-support tools and require careful validation to avoid digital exclusion, privacy risks, and algorithmic bias [47, 48].

## Conclusion

Women with advanced or metastatic breast cancer reported substantially higher financial burden than the female German reference population. Lower HRQoL, younger age, and living alone were associated with greater financial impact. These findings support routine assessment of financial distress in patients with advanced breast cancer and suggest that PRO- or ePRO-based screening may help identify patients in need of early social-medical support. Future studies should evaluate digital supportive-care pathways that combine financial distress screening with HRQoL, symptom burden, and time toxicity to better capture the multidimensional burden of advanced cancer care.

## Data Availability

No datasets were generated or analyzed during the current study.

## References

[1] Shang C, Xu D (2022) Epidemiology of breast cancer. Oncologie 24(4):649–663. 10.32604/oncologie.2022.027640

[2] Robert-Koch-Institut. Breast cancer: incidence. Berlin 2025. URL: https://www.gbe.rki.de/EN/Topics/HealthStatus/PhysicalHealth/Cancer/BreastCancerIncidence/BreastCancerIncidence_node.html?darstellung=0&kennzahl=1&zeit=2023&geschlecht=0&standardisierung=3 (accessed on 28 April 2026).

[3] Voeltz D, Baginski K, Hornberg C, Hoyer A (2024) Trends in incidence and mortality of early-onset cancer in Germany between 1999 and 2019. Eur J Epidemiol 39(7):827–837. 10.1007/s10654-024-01134-4 38819553 10.1007/s10654-024-01134-4 PMC11343808

[4] Hübner J, Katalinic A, Waldmann A, Kraywinkel K (2020) Long-term incidence and mortality trends for breast cancer in Germany. Geburtshilfe Frauenheilkd 80(6):611–618. 10.1055/a-1160-5569 32565551 10.1055/a-1160-5569 PMC7299687

[5] Katalinic A, Eisemann N, Kraywinkel K, Noftz MR, Hübner J (2020) Breast cancer incidence and mortality before and after implementation of the German mammography screening program. Int J Cancer 147(3):709–718. 10.1002/ijc.32767 31675126 10.1002/ijc.32767

[6] Łukasiewicz S, Czeczelewski M, Forma A, Baj J, Sitarz R, Stanisławek A (2021) Breast cancer-epidemiology, risk factors, classification, prognostic markers, and current treatment strategies-an updated review. Cancers (Basel) 13(17):4287. 10.3390/cancers13174287 34503097 10.3390/cancers13174287 PMC8428369

[7] Roheel A, Khan A, Anwar F, Akbar Z, Akhtar MF, Imran Khan M, Sohail MF, Ahmad R (2023) Global epidemiology of breast cancer based on risk factors: a systematic review. Front Oncol 13:1240098. 10.3389/fonc.2023.1240098 37886170 10.3389/fonc.2023.1240098 PMC10598331

[8] Johansson ALV, Stensheim H (2020) Epidemiology of pregnancy-associated breast cancer. Adv Exp Med Biol 1252:75–79. 10.1007/978-3-030-41596-9_9 32816264 10.1007/978-3-030-41596-9_9

[9] Simoes E, Graf J, Sokolov AN, Grischke EM, Hartkopf AD, Hahn M, Weiss M, Abele H, Seeger H, Brucker SY (2018) Pregnancy-associated breast cancer: maternal breast cancer survival over 10 years and obstetrical outcome at a university centre of women’s health. Arch Gynecol Obstet 298(2):363–372. 10.1007/s00404-018-4822-5 29931523 10.1007/s00404-018-4822-5

[10] Tauber N, Amann N, Dannehl D, Deutsch TM, Dimpfl M, Fasching P, Hartkopf A, Heublein S, Hilmer L, Hörner M, Krawczyk N, Krückel A, Krug D, Marmé F, Michel LL, Reinisch M, Rody A, Schäffler H, Schneeweiss A, Utz D, Veselinovic K, Banys-Paluchowski M (2025) Therapy of early breast cancer: current status and perspectives. Arch Gynecol Obstet 312(2):311–328. 10.1007/s00404-025-08028-0 40261372 10.1007/s00404-025-08028-0 PMC12334469

[11] National Cancer Institute. Breast Cancer Prognosis and Survival Rates. U.S. Department of Health and Human Services 2025. URL: https://www.cancer.gov/types/breast/survival (accessed on 28 April 2026).

[12] American Cancer Society. Survival Rates for Breast Cancer. 2026. URL: https://www.cancer.org/cancer/types/breast-cancer/understanding-a-breast-cancer-diagnosis/breast-cancer-survival-rates.html (accessed on 28 April 2026).

[13] Michel LL, Feisst M, Thewes V, Jäger D, Hartkopf AD, Brucker SY, Uhrig S, Ziegler P, Beckmann MW, Belleville E, Maurer C, Fasching PA, Smetanay K, Fremd C, Schneeweiss A (2014) Overall survival in metastatic breast cancer patients: real-world data from 1,000 patients treated at the NCT Heidelberg between 2014 and 2022. Breast Care (Basel). 10.1159/000548610 10.1159/000548610 PMC12618037 41244618

[14] Wallwiener M, Simoes E, Sokolov AN, Brucker SY, Fasching PA, Graf J (2016) Health-related quality of life in metastatic and adjuvant breast cancer patients. Geburtshilfe Frauenheilkd 76(10):1065–1073. 10.1055/s-0042-113188 27761027 10.1055/s-0042-113188 PMC5065420

[15] Simoes E, Sokolov AN, Graf J, Pavlova MA, Brucker SY, Wallwiener D, Schmahl FW, Bamberg M (2016) Why strive after clinical social medicine? From epidemiological association to personalized social medicine a Case of Breast Cancer Care. J Gesundheitswesen. 8(2):97–102. 10.1055/s-0042-100822 10.1055/s-0042-100822 26906534

[16] Sesto ME, Carroll CB, Zhang X et al (2022) Unmet needs and problems related to employment and working as reported by survivors with metastatic breast cancer. Support Care Cancer 30(5):4291–4301. 10.1007/s00520-021-06755-z 35088147 10.1007/s00520-021-06755-z PMC8959021

[17] Coughlin SS (2019) Social determinants of breast cancer risk, stage, and survival. Breast Cancer Res Treat 177(3):537–548. 10.1007/s10549-019-05340-7 31270761 10.1007/s10549-019-05340-7

[18] Wiese D, Stroup AM, Crosbie A, Lynch SM, Henry KA (2019) The impact of neighborhood economic and racial inequalities on the spatial variation of breast cancer survival in New Jersey. Cancer Epidemiol Biomarkers Prev 28(12):1958–1967. 10.1158/1055-9965.EPI-19-0416 31649136 10.1158/1055-9965.EPI-19-0416

[19] Nattinger AB, Wozniak EM, McGinley EL, Li J, Laud P, Pezzin LE (2017) Socioeconomic disparities in mortality among women with incident breast cancer before and after implementation of Medicare Part D. Med Care 55(5):463–469. 10.1097/MLR.0000000000000685 28030476 10.1097/MLR.0000000000000685 PMC5391268

[20] Jansen L, Eberle A, Emrich K, Gondos A, Holleczek B, Kajüter H, Maier W, Nennecke A, Pritzkuleit R, Brenner H, GEKID Cancer Survival Working Group (2014) Socioeconomic deprivation and cancer survival in Germany an ecological analysis in districts in Germany. Int J Cancer 134(12):2951–2960. 10.1002/ijc.28624 24259308 10.1002/ijc.28624

[21] O’Donnell O (2024) Health and health system effects on poverty: a narrative review of global evidence. Health Policy 142:105018. 10.1016/j.healthpol.2024.105018 38382426 10.1016/j.healthpol.2024.105018

[22] Anser MK, Yousaf Z, Khan MA, Nassani AA, Alotaibi SM, Qazi Abro MM, Vo XV, Zaman K (2020) Does communicable diseases (including COVID-19) may increase global poverty risk? A cloud on the horizon. Environ Res 187:109668. 10.1016/j.envres.2020.109668 32422482 10.1016/j.envres.2020.109668 PMC7228701

[23] Xu L, Guo M, Nicholas S, Sun L, Yang F, Wang J (2020) Disease causing poverty: adapting the Onyx and Bullen social capital measurement tool for China. BMC Public Health 20(1):63. 10.1186/s12889-020-8163-5 31937283 10.1186/s12889-020-8163-5 PMC6961236

[24] Jiahui L, Xingfeng L, Lijie W et al (2025) Breast cancer patients’ experiences of coping with financial toxicity: a systematic review and qualitative meta-synthesis. Psychooncology 34(1):e70075. 10.1002/pon.70075 39817747 10.1002/pon.70075 PMC11737291

[25] Koch L, Jansen L, Herrmann A, Stegmaier C, Holleczek B, Singer S, Brenner H, Arndt V (2013) Quality of life in long-term breast cancer survivors - a 10-year longitudinal population-based study. Acta Oncol 52(6):1119–1128. 10.3109/0284186X.2013.774461 23514583 10.3109/0284186X.2013.774461

[26] Hildenbrand S, Graf J, Michaelis M, Wagner A, Völter-Mahlknecht S, Simoes E, Rieger MA (2025) Evaluation of social medical skills’ relevance in the study course from the medical students’ perspective: a retrospective trend study. Educ Sci 15(10):1408. 10.3390/educsci15101408

[27] Graf J, Simoes E, Wißlicen K, Rava L, Walter CB, Hartkopf A, Keilmann L, Taran A, Wallwiener S, Fasching P, Brucker SY, Wallwiener M (2016) Willingness of patients with breast cancer in the adjuvant and metastatic setting to use electronic surveys (ePRO) depends on sociodemographic factors, health-related quality of life, disease status and computer skills. Geburtshilfe Frauenheilkd 76(5):535–541. 10.1055/s-0042-105872 27239062 10.1055/s-0042-105872 PMC4873300

[28] Hartkopf AD, Graf J, Simoes E, Keilmann L, Sickenberger N, Gass P, Wallwiener D, Matthies L, Taran FA, Lux MP, Wallwiener S, Belleville E, Sohn C, Fasching PA, Schneeweiss A, Brucker SY, Wallwiener M (2017) Electronic-based patient-reported outcomes: willingness, needs, and barriers in adjuvant and metastatic breast cancer patients. JMIR Cancer 3(2):e11. 10.2196/cancer.6996 28784595 10.2196/cancer.6996 PMC5565790

[29] Graf J, Sickenberger N, Brusniak K, Matthies LM, Deutsch TM, Simoes E, Plappert C, Keilmann L, Hartkopf A, Walter CB, Hahn M, Engler T, Wallwiener S, Schuetz F, Fasching PA, Schneeweiss A, Brucker SY, Wallwiener M (2022) Implementation of an electronic patient-reported outcome app for health-related quality of life in breast cancer patients: evaluation and acceptability analysis in a two-center prospective trial. J Med Internet Res 24(2):e16128. 10.2196/16128 35133288 10.2196/16128 PMC8864528

[30] Clarke NA, Braverman J, Worthy G, Shaw JW, Bennett B, Dhanda D, Cocks K (2024) A review of meaningful change thresholds for EORTC QLQ-C30 and FACT-G within oncology. Value Health 27(4):458–468. 10.1016/j.jval.2023.12.012 38191023 10.1016/j.jval.2023.12.012

[31] EORTC Quality of Life Group. EORTC QLQ-C30, Version 3 in German. 1995. URL: https://www.eortc.be/qol/C30/QLQ-C30%20German.pdf (accessed on 29 April 2026).

[32] EORTC Data Center. EORTC QLQ-C30 Scoring Manual. Brussels 2001. URL: https://www.eortc.org/app/uploads/sites/2/2018/02/SCmanual.pdf (accessed on 29 April 2026).

[33] Hinz A, Singer S, Brähler E (2014) European reference values for the quality of life questionnaire EORTC QLQ-C30: results of a German investigation and a summarizing analysis of six European general population normative studies. Acta Oncol 53(7):958–965. 10.3109/0284186X.2013.879998 24456505 10.3109/0284186X.2013.879998

[34] Cohen J (1992) A power primer. Psychol Bull 112(1):155–159 19565683 10.1037//0033-2909.112.1.155

[35] Cook R, Weisberg S (1982) Criticism and influence analysis in regression. Sociol Methodol 13:313–361

[36] Yin J, Wang C, Hou Y, Cai G, Song X, Qin C (2026) Financial toxicity among patients with breast cancer: a systematic review and meta-analysis. Oncologist 31(2):oyaf417. 10.1093/oncolo/oyaf417 41397911 10.1093/oncolo/oyaf417 PMC12831935

[37] Ehsan AN, Wu CA, Minasian A, Singh T, Bass M, Pace L, Ibbotson GC, Bempong-Ahun N, Pusic A, Scott JW, Mekary RA, Ranganathan K (2023) Financial toxicity among patients with breast cancer worldwide: a systematic review and meta-analysis. JAMA Netw Open 6(2):e2255388. 10.1001/jamanetworkopen.2022.55388 36753274 10.1001/jamanetworkopen.2022.55388 PMC9909501

[38] Vancoppenolle J, Franzen N, Azarang L, Juslin T, Krini M, Lubbers T, Mattson J, Mayeur D, Menezes R, Schmitt J, Scotte F, Seoane López O, Skaali T, Ubels J, Schlander M, Retel V, van Harten WH, OECI Working Group Health Economics (2025) Financial toxicity and socioeconomic impact of cancer in Europe. ESMO Open 10(6):105293. 10.1016/j.esmoop.2025.105293 40494040 10.1016/j.esmoop.2025.105293 PMC12180988

[39] Fabian A, Domschikowski J, Greiner W, Bockelmann G, Karsten E, Rühle A, Nicolay NH, Grosu AL, Dunst J, Krug D (2022) Financial toxicity in cancer patients treated with radiotherapy in Germany-a cross-sectional study. Strahlenther Onkol 198:1053–1061. 10.1007/s00066-022-01936-z 35467099 10.1007/s00066-022-01936-z PMC9700565

[40] Tetzlaff BO, Rühle A, Domschikowski J, Trommer M, Ferdinandus S, Becker JN, Wurschi G, Böke S, Grott CA, Käsmann L, Schneider M, Bockelmann E, Krug D, Nicolay NH, Fabian A, Sonnhoff M (2026) Financial toxicity in breast cancer patients during radiotherapy - A German multicenter analysis. Cancer Treat Res Commun 46:101071. 10.1016/j.ctarc.2025.101071 41401706 10.1016/j.ctarc.2025.101071

[41] Riccetti N, Felberbaum R, Flock F, Kühn T, Leinert E, Schwentner L, Singer S, Taylor K, Wöckel A, Janni W (2022) Financial difficulties in breast cancer survivors with and without migration background in Germany-results from the prospective multicentre cohort study BRENDA II. Support Care Cancer 30(8):6677–6688. 10.1007/s00520-022-07074-7 35507113 10.1007/s00520-022-07074-7 PMC9213307

[42] Perrone F, Jommi C, Di Maio M, Gimigliano A, Gridelli C, Pignata S, Ciardiello F, Nuzzo F, de Matteis A, Del Mastro L, Bryce J, Daniele G, Morabito A, Piccirillo MC, Rocco G, Guizzaro L, Gallo C (2016) The association of financial difficulties with clinical outcomes in cancer patients: secondary analysis of 16 academic prospective clinical trials conducted in Italy. Ann Oncol 27(12):2224–2229. 10.1093/annonc/mdw433 27789469 10.1093/annonc/mdw433

[43] Romeu Gordo L, Simonson J, Lozano Alcántara A (2024) Financial consequences of COVID-19 in Germany: living standards of older people during the first year of the pandemic. J Aging Soc Policy 36(6):1567–1584. 10.1080/08959420.2023.2257535 37732559 10.1080/08959420.2023.2257535

[44] Bundesministerium für Gesundheit. Entwurf eines Gesetzes zur Stabilisierung der Beitragssätze in der gesetzlichen Krankenversicherung (GKV-Beitragssatzstabilisierungsgesetz). Referentenentwurf. Berlin 2026. URL: https://www.bundesgesundheitsministerium.de/fileadmin/Dateien/3_Downloads/Gesetze_und_Verordnungen/GuV/S/RefE_BStabG_2026.pdf (accessed on 30 April 2026).

[45] Techniker Krankenkasse. Gesundheitsreport Arbeitsunfähigkeiten. Hamburg 2025. URL: https://www.tk.de/resource/blob/2194002/828793b4b4a5953abece5e4874ce79b9/gesundheitsreport-au-2025-data.pdf (accessed on 30 April 2026).

[46] Bundesministerium der Justiz und für Verbraucherschutz. Sozialgesetzbuch (SGB) Fünftes Buch (V) - Gesetzliche Krankenversicherung - (Artikel 1 des Gesetzes v. 20. Dezember 1988, BGBl. I S. 2477). URL: https://www.gesetze-im-internet.de/sgb_5/BJNR024820988.html (accessed on 30 April 2026).

[47] Mudaranthakam DP, Makovec A, Forcino R et al (2025) A hybrid technology-enabled financial navigation model to combat financial toxicity in cancer care. Cancer Control 32:10732748251387383. 10.1177/10732748251387383 41062166 10.1177/10732748251387383 PMC12511694

[48] Kaufmann TL, Rocque GB (2024) Using electronic patient-reported outcome monitoring to navigate patients to supportive care services. JCO Oncol Pract 20(10):1297–1299. 10.1200/OP.24.00270 38917403 10.1200/OP.24.00270 PMC13040481

